# The Growth of the Nursing Profession: IV. Some Economic and Practical Aspects

**Published:** 1918-02-23

**Authors:** 


### THE MATRONS' AND SISTERS' DEPARTMENT.

### THE GROWTH OF THE NURSING PROFESSION.

### IV. Some Economic and Practical Aspects.

In carrying out its task of reorganising the profession, the College of Nursing, as already indicated, will have to pay due attention to the economic aspect thereof. This is a large question, and one which involves vested interests to a greater extent than any educational consideration. There is, however, ground for hope, for the limelight which has been playing upon nurses and their doings during the past three years of war has revealed the inadequacy of their remuneration. Hospital authorities are beginning to realise that the salaries which they offered in times past are nowadays little short of an insult to the class of woman whom they wish to secure, and are altering their scale in accordance. A piecemeal reform of this sort, although welcome as an indication of an awakened conscience, is not, however, really sufficient. What is needed is that the public—not the charitable public, but the thinking professional and commercial public—should realise why it is necessary that nurses' payments should in future be on a different footing. This is not a matter of sentiment, it is a matter of business. The number of ways in which a woman can earn her living are now as many, and offer as varied attractions, as the callings open to men. It is said that nurses are born, not made. But many "born nurses" are not aware of the fact until they have been trained, and there are many women now in other walks of life who, if they were trained, might well turn out to be "born nurses."

It is, therefore, a matter of sound business to make the profession of nursing one which will attract not only those who are moved thereto by a strong impulse, but also those who have in them the makings of good nurses. These latter, looking round on the careers open to them, will in most cases be compelled to consider the financial future offered by each career. If the nursing profession as a whole remains much less remunerative than other callings, then it cannot hope to reach the standard of quality and quantity which is not only desirable, but is absolutely essential, in the interests of the health of the community. Now this enlightening of the right people is a task which can best be carried out by or in connection with a representative body of the whole profession. An array of facts and figures, proceeding from an individual or a small organisation, may startle and stir a few active minds here and there. But if the same facts and figures are taken in hand by a representative body which has the weight of the profession at its back, they can be so used as to bring home their significance to a far larger and more important audience—more important because better placed to secure reform. If the rank and file of British nurses would once realise that by joining the College of Nursing they are thus taking a direct step towards bringing about reforms which concern them intimately, they would join without any further hesitation.

While insisting on the necessity of an adequate remuneration for all classes of nursing service, it must at the same time be borne in mind that nurses themselves will be the first to realise the inherent limitations of any improvement in this direction, and will not expect to make a fortune out of their profession. Out of the average salary, even on a revised scale, a nurse by skimping and saving and self-denial may contrive to put by sufficient to enable her to live when her working days are over. But if sickness or unforeseen distress intervenes, then resort must be had to these savings, and the future once more looms threateningly in her mind. Now self-denial and thrift are excellent things, and greatly to be encouraged in nurses, but as virtues they have their limitations. A nurse should always be able to give the fullest and best service of which she is capable. If to her mental vision that future rainy day is so insistently present that in order to meet it she denies herself not only what the average person would call legitimate pleasures, but also books and reading and general physical and mental recreation, then she will not be able to give her best to her patients. The provision of a Benevolent Fund for the nursing profession, for the benefit of nurses who through no fault of their own have not been able to provide for sickness and old age, is therefore not a question of charity or sentiment. It is a question of the public welfare, and everyone who helps in the establishment of such a fund is contributing not to charity, but to increased efficiency in the nursing services.

